# Kinetic Isotope Effects and Hydrogen Tunnelling in PCET Oxidations of Ascorbate: New Insights into Aqueous Chemistry?

**DOI:** 10.3390/molecules25061443

**Published:** 2020-03-23

**Authors:** Ana Karković Marković, Cvijeta Jakobušić Brala, Viktor Pilepić, Stanko Uršić

**Affiliations:** Faculty of Pharmacy and Biochemistry, University of Zagreb, A. Kovačića 1, 10 000 Zagreb, Croatia; akarkovic@pharma.hr (A.K.M.); vpilepic@pharma.hr (V.P.)

**Keywords:** kinetic isotope effects, ascorbate PCET reactions, hydrogen tunnelling, water vibrational dynamics, activation processes

## Abstract

Recent experimental studies of kinetic isotope effects (KIE-s) and hydrogen tunnelling comprising three proton-coupled electron transfer (PCET) oxidations of ascorbate monoanion, (a) in aqueous reaction solutions, (b) in the mixed water-organic cosolvent systems, (c) in aqueous solutions of various salts and (d) in fairly diluted aqueous solutions of the various partial hydrophobes are reviewed. A number of new insights into the wealth of the kinetic isotope phenomena in the PCET reactions have been obtained. The modulation of KIE-s and hydrogen tunnelling observed when partially hydrophobic solutes are added into water reaction solution, in the case of fairly diluted solutions is revealed as the strong linear correlation of the isotopic ratios of the Arrhenius prefactors *A*h/*A*d and the isotopic differences in activation energies Δ*E_a_* (D,H). The observation has been proposed to be a signature of the involvement of the collective intermolecular excitonic vibrational dynamics of water in activation processes and aqueous chemistry.

## 1. Introduction

The aim of this review is to present the main results of our recent experimental studies of kinetic isotope effects (KIE-s) and hydrogen tunnelling in some proton-coupled electron transfer (PCET) reactions of ascorbate monoanion in aqueous reaction solutions and in the mixed water-organic cosolvent systems. The issues of KIE-s and hydrogen tunnelling [1,2,3,4,5,6,7,8,9] and PCET reactions [10,11,12,13,14,15] are well elaborated in many relevant books and publications. The phenomenon of PCET [10,11,12,13,14,15] is ubiquitous throughout chemistry and biology. Hence, the study of KIE-s in PCET reactions is of importance not only for understanding PCET reactions in solution; above all, many enzymatic reactions involve PCET as well. However, to the best of our knowledge, there are no systematic studies of solvent influences on KIE-s and hydrogen tunnelling in PCET reactions [16]. We have investigated KIE-s and hydrogen tunnelling in the following PCET oxidations of ascorbate: (a) reaction with 2,2,6,6-tetramethylpiperidine-1-oxyl radical (TEMPO radical) [17,18], (b) reaction with hexacyanoferrate(III) ions [19,20,21,22] and (c) reaction with ferricinium cation [22] in aqueous solutions containing substantial concentrations of organic solvents or salts. Furthermore, KIE-s and hydrogen tunnelling in reaction of ascorbate with hexacyanoferrate(III) ions have been investigated in a series of fairly diluted aqueous solutions containing small concentrations of the partially hydrophobic solutes [23]. These studies gave some new and surprising insights about KIE-s in the PCET reactions. Moreover, the studies could be in support of the recently proposed [24,25,26,27] involvement of the vibrational water dynamics [24,25,26,27,28,29,30,31,32,33] in activation processes in aqueous chemistry.

## 2. Kinetic Isotope Effects and Hydrogen Tunnelling in Proton-Coupled Electron Transfer Reactions

### 2.1. Proton-Coupled Electron Transfer Reactions

Proton-coupled electron transfer, as generally accepted today, is broadly defined as any process which includes a transfer of an electron and a proton. In these reactions, an electron and a proton can be transferred between the same or different sites, in the same or in different directions, by concerted or stepwise mechanisms. PCET also includes processes in which protons modulate the electron transfer process even if they themselves do not transfer [10,11,12,13]. The term PCET was introduced for the first time by T. J. Meyer in 1981, for reactions of ruthenium-oxo complexes, where a proton and electron are transferred in a single, concerted step. Throughout this review, we discuss concerted PCET reactions, denoted as PCET. In concerted PCET reactions the electron and proton transfer “together”, in a single kinetic step, thus circumventing high energy intermediates which would be formed in the case of stepwise mechanism [34]. Reactions in which the electron and proton transfer between the same sites are denoted as hydrogen atom transfer (HAT), and between different sites as multiple-site concerted proton-electron transfer (MS-CPET) [12].

Concerted PCET is an important mechanism for charge transfer in a wide variety of biological processes, such as photosynthesis, respiration and many enzymatic reactions, as well as chemical and electrochemical processes, particularly those relevant to solar cells and other energy devices [7,10,11,12,13,14,35,36,37,38,39,40,41,42,43]. The long-distance transfer of electrons in biology often exhibits the characteristics of PCET. Inspired by the photosynthetic photosystem II, which utilizes solar energy and catalyses water splitting reaction, a variety of solar energy devices have been developed. One of the first developed molecular catalysts was Ru “blue dimer” [10]. Today, great attention is directed toward the design of various metal-based catalysts, electrocatalysts, multiproton relays, nanoparticle catalysts [14,44]. One example is Dye Sensitized Photoelectrosynthesis Cell which combines the properties of wide bandgap semiconductor nanoparticle oxides and the light absorption/catalytic properties of the chromophore–catalyst [45]. Recently, it was observed that excited-state PCET reactions can access the Marcus inverted region, which is of great importance in the development of solar energy technologies [15,46,47]. In addition, it opened the intriguing question about the role of inverted region in biological pathways [48]. Moreover, the applications of PCET in organic synthesis and drug discovery have attracted great attention in recent years [38,49,50,51,52]. PCET reaction enabled a new route in the production of ammonia, one of the most important industrial processes [53]. In addition to these complex processes, the investigation of simpler model systems is also important for investigating the fundamental physical principles underlying PCET reactions [10,11,12]. Recently, the first examples of MS-CPET for the activation and formation of C−H bonds have been reported [12,49,50].

To understand complex physical properties of PCET reactions, a combined experimental and theoretical approach is essential. The theory for PCET reactions mostly developed by Hammes-Schiffer group combines the concepts from Marcus theory for electron transfer and analogous theories for proton transfer [13,34,35,54]. The theory describes PCET reaction in terms of vibronically nonadiabatic transitions including the effects of thermal fluctuations of the solvent or protein environment, and the proton donor-acceptor motion. The theory differentiates between HAT and MS-CPET regarding the degree of electronic nonadiabaticity, where HAT corresponds to electronically adiabatic and MS-CPET to electronically nonadiabatic proton transfer. Within the framework of the theory, a series of rate constant expressions have been derived for PCET reactions in different regimes. The simplest expression for rate constant of vibronically nonadiabatic PCET and electronically nonadiabatic proton transfer, is given with Equation (1):

(1) $$k=\underset{\mu }{\sum }P_{\mu }\underset{\nu }{\sum }\frac{{\left|V^{\mathrm{el}}S_{\mu \nu }\right|}^{2}}{\hbar }\sqrt{\frac{\pi }{\lambda k_{B}T}}\exp\left[-\frac{{\left(\Delta G_{\mu \nu }^{0}+\lambda \right)}^{2}}{4\lambda k_{B}T}\right]$$

where *μ* and *ν* are reactant and product vibronic states, *P_μ_* is the Boltzmann probability, *V*^el^ is the electronic coupling, *S_μν_* is the overlap between the reactant and product vibrational wavefunctions, *λ* is the reorganization energy, ΔGº*_μν_* is the reaction free energy.

The equation is expanded by including the proton donor-acceptor motion, *R*-mode, characterized by oscillator’s effective mass, *M* and frequency, *Ω*. Rate expressions in the low-frequency (high-temperature) regime for *R*-mode, where the energy associated with the *R*-mode is lower than the thermal energy, ℏ*Ω* ≪ *k*_B_*T*, and in high-frequency (low-temperature) regime, for ℏ*Ω* ≫ *k*_B_*T*, have been derived. Rate expression in low-frequency regime is identical to the rate expression derived by Kutznetsov and Ulstrup and implemented by Klinman and others in the study of enzymatic PCET reactions [8].

### 2.2. Kinetic Isotope Effects in PCET Reactions

Kinetic isotope effect, the change of rate that occurs upon isotopic substitution, is a widely used tool for elucidating a reaction mechanism, particularly in the study of the hydrogen (H^+^, H^−^, H•) transfer reactions and the role of hydrogen tunnelling (see Section 2.3.) [1,2,3,4,5,6,55]. Therefore, the analysis of KIE reveals also the fundamental features of PCET reactions [4,6,9,10,11,12,13,14,47,56,57,58,59]. KIE can be used to distinguish between concerted and stepwise PCET reactions. The experimentally observed magnitudes of the KIE in PCET reactions vary widely, from KIE < 2 to very large and even inverse values [10,11,60]. KIE of 13,000 was observed in the PCET reaction of the isomerization of 2,4,6-tri-tert-butylphenyl at low temperature [61]. In PCET reduction of benzoquinone to hydroquinone by various osmium complexes “colossal” KIE-s from 180 to 460 were observed [62,63]. Moreover, in PCET reaction catalysed by enzyme soybean lipoxygenase (SLO), wild type KIE of ~ 80 [64] increased to the values from 553 to 760 in the case of various mutants [7,41,65,66].

Recently, KIE has been used in the study of the excited-state PCET in DNA duplex [67], in concerted proton-coupled interfacial electron transfer [44], and heterogeneous electrochemical PCET reaction [68]. In addition, it has been widely used in the study of enzymatic reactions, many of which are PCET reactions [7,55,64,65,66,69,70]. The temperature-dependences of KIE in PCET reactions vary from significantly temperature-dependent, to temperature-independent KIE-s [7,19,20,69,70,71]. This wide span of KIE temperature-dependences, observed in a great number of enzymatic but also in solution PCET reactions are usually interpreted with Marcus like full-tunnelling model (see Section 2.3.) [7,64,72]. In the case of PCET ubiquinol oxidation by cytochrome *bc*1 even inverse temperature dependence was observed [60].

Within PCET theory (see Section 2.1.) expressions for KIE in two *R-*mode regimes were derived [13,73]. In low-frequency regime, with assumptions that only ground states contribute to the rate, and reorganization energy and driving force are independent of the isotope, the KIE is given with Equation (2):

(2) $$\mathrm{KIE}\approx \;\frac{{\left|S_{H}\right|}^{2}}{{\left|S_{D}\right|}^{2}}\exp\left[-\frac{2k_{B}T}{M\Omega^{2}}\left(\alpha_{D}^{2}-\alpha_{H}^{2}\right)\right]$$

where *S* is the overlap between the reactant and product vibrational wavefunctions, *M* and *Ω* are effective mass and frequency of proton donor-acceptor oscillator, *α* represents exponential dependence of overlap on proton donor-acceptor distance, *R.*

The magnitude of the KIE primarily depends on distance and frequency. KIE increases as *R* increases, if all other parameters are constant, due to the H/D difference in distance dependence of proton vibrational wavefunctions overlap. KIE increases as *Ω* increases since higher frequency typically does not enable effective sampling of smaller distances. Very often, a decrease of *R* leads to an increase of *Ω* leading to the complex dependence of KIE on *R*. Contributions from excited vibronic states lead to a decrease of the KIE due to the greater overlap of excited states. Moreover, excited states contribute more to the deuterium overall rate, because of the smaller splittings between the energy levels. The theory also predicts the dependence of KIE on temperature, driving force and solvent reorganization energy. KIE decreases with increasing temperature due to the greater contribution of excited states and the temperature-dependence of KIE will increase as the frequency *Ω* decreases. KIE has the greatest value near zero driving force and decreases as reactions are more endergonic or exergonic, due to increased contributions from excited vibronic states, and also could even become inverse in the inverted region [46]. KIE is relatively insensitive to changes in the solvent reorganization energy in the 10−40 kcal/mol range [10,73].

In low-temperature (high-frequency) regime for the *R*-mode, as in the case of a rigid hydrogen bond, KIE is given with Equation (3):

(3) $$\mathrm{KIE}=\frac{S_{H}^{2}}{S_{D}^{2}}\exp\left[-\frac{\hbar \left(\alpha_{D}^{2}-\alpha_{H}^{2}\right)}{2M\Omega }\right]$$

KIE increases as *R* and *Ω* increase, and is independent of the driving force and temperature when only the ground states contribute to the rate. Contributions from excited states could lead to either a decrease or an increase of the KIE with temperature, as observed experimentally in the case of ubiquinol analog oxidation [74].

Recently, reactive mode composition factor method has been applied to predict KIE and hydrogen tunnelling in a series of PCET reactions [56].

### 2.3. Hydrogen Tunnelling

**Figure 1:**
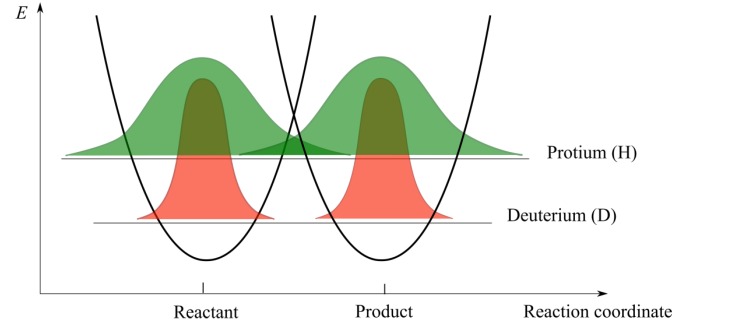
Illustration of the ground-state quantum mechanical tunnelling of hydrogen. Black parabolas represent potential energy (*E*) curves for reactant and product. The wavefunction of lighter isotope (protium, in green) is less localized due to a smaller mass in comparison to heavier isotope (deuterium, in red). Overlap of reactant’s and product’s hydrogen wavefunctions can lead to H-tunnelling.

Hydrogen (H^+^, H^−^, H•) transfer is one of the most ubiquitous reactions that take place in nature, especially among enzyme catalysed reactions [7,8,69,75,76]. Hydrogen has the small mass and de Broglie wavelength of similar magnitude to molecular dimensions or distances expected for hydrogen transfer to occur. Therefore, the quantum mechanical behaviour of hydrogen particle like hydrogen tunnelling could be expected to play a significant role in hydrogen transfer reactions [3,7,69,77]. Quantum mechanical tunnelling of hydrogen can occur when the wavefunction of hydrogen on the reactant (hydrogen donor) overlaps with that of the hydrogen on the product (hydrogen acceptor) (Figure 1).

Tunnelling is very sensitive to the shape (width and height) of the barrier; it can happen well below the barrier maximum in the case of a narrow barrier or close to the top in the case of a broad barrier [70,78]. The early theoretical model for hydrogen tunnelling developed by Bell was based on tunnelling correction of the semi-classical rate constant within a framework of the transition-state theory [3]. The common signatures of hydrogen tunnelling according to Bell model were the values of isotopic Arrhenius prefactor ratios *A*h/*A*d and the isotopic differences in activation enthalpies ΔΔ*H*^‡^ that are well beyond the semiclassical limits [3,5,6]. Although Bell model could explain large and temperature-dependent KIE-s, it failed to explain the temperature-independent KIE-s under room temperature conditions observed in some enzymatic H-transfer reactions [7,8,69,76,79]. Models that can accommodate both temperature-dependent and temperature-independent KIE near room temperature are full tunnelling models, also known as Marcus-like models [7,69,76,78]. Within this approach, the motion of heavy atoms of donor and acceptor is treated quantum-mechanically. Reorganization of heavy atoms leads to a tunnelling-ready state with transiently degenerate potential energy surfaces of reactants and products that allow for donor-acceptor wavefunctions to overlap. The effect of donor-acceptor distance (DAD) in tunnelling ready state is thoroughly investigated in many enzymatic C–H bond cleavage reactions [7,69,76]. The crucial experimental parameter for H-tunnelling that emerged from such studies is the difference between energies of activation for hydrogen isotopes H and D, Δ*E_a_* = *E*_a_(D) − *E*_a_(H), i.e., the temperature dependence of KIE. In many cases, for the most active wild-type enzymes Δ*E*_a_ is nearly 0 (temperature-independent KIE) suggesting that DAD is sufficiently short to ensure effective tunnelling of both H and D isotopes. Impaired enzymes show enlarged values of Δ*E*_a_ (temperature-dependent KIE) and poor overlap of deuterium wavefunctions that necessitate further DAD sampling to achieve adequate DAD for tunnelling. This experimentally and theoretically predicted behaviour was recently confirmed and corroborated by high-precision electron−nuclear double resonance (ENDOR) spectroscopy of prototypical nonadiabatic tunnelling system, soybean lipoxygenase [80].

## 3. Kinetic Isotope Effects and Hydrogen Tunnelling in Ascorbate PCET Oxidations

Kinetic isotope effects and hydrogen tunnelling (recall that the transfer of proton and electron in concerted PCET corresponds to “net” hydrogen transfer) have been investigated in the PCET ascorbate oxidations comprising three different reactions and three different cases with respect to the charge bearing the oxidants: 2,2,6,6-tetramethylpiperidine-1-oxyl radical (TEMPO) has no net charge, hexacyanoferrate(III) is negatively and ferricinium ion is positively charged. The choice of the oxidants bearing different charges should also, be related to the donor-acceptor distance (DAD) in a transition configuration in PCET reaction (due to repulsive/attractive forces between the redox partners) since DAD is of great importance for the magnitude of KIE in the reaction [10,13,35,54,73]. Furthermore, the effective barrier width, the crucial factor determining probability of quantum mechanical tunnelling can be in relation to sampling of optimal donor-acceptor distances at the tunnelling-ready state. In the cases examined, repulsive/attractive forces that appear due to charge on the reactants (or are absent when a reactant is uncharged) would influence DAD in a different manner which could be, among others, of significance for the magnitude of DAD in the interaction with negatively charged ascorbate monoanion.

The kinetic isotope effects observed in almost all ascorbate PCET systems presented here are increased unexpectedly [17,18,19,21,22,23] with regard to the KIE-s observed in particular “neat” water reaction. The only exception is the case of hexacyanoferrate oxidant in more concentrated water solutions of inorganic salts [20] where the observed KIE-s are reduced in magnitude.

The most common phenomenon observed throughout all the three investigated PCET reactions of ascorbate including all the solvent/solute added systems, regardless of the organic cosolvent or the salt added in the water reaction solution is the appearance of hydrogen tunnelling. The observed common signatures of hydrogen tunnelling in a reaction, the values of isotopic Arrhenius prefactor ratios *A*h/*A*d and the isotopic differences in *E_a_*(D,H) or activation enthalpies ΔΔ*H*^‡^(D,H) that are well beyond semiclassical limits [3,5,6] suggest so-called moderate hydrogen tunnelling in all the cases excepting the more concentrated water solutions of the inorganic salts (keeping in mind, however, that *E_a_*(D,H) and ΔΔ*H*^‡(^(D,H) are not entirely equivalent). In these systems, values of the isotopic differences in activation enthalpies ΔΔ*H*^‡^(D,H) below the semiclassical limit of 5.1 kJ/mol and the isotopic prefactor ratios *A*h/*A*d above the upper semiclassical limit of 1.4, indicative of entering into extensive tunnelling where both the isotopes tunnel were observed.

Seemingly, in the initial system, the reaction in “neat” water, there could be no obvious tunnelling manifested. However, taking into account the great KIE that is well beyond the semiclassical limits observed in the interaction of ascorbate monoanion and the TEMPO radical in water reaction system [17,18], as well as the ΔΔ*H*^‡^(D,H) observed in the case, tunnelling should occur in the reaction. The case of interaction of ascorbate with hexacyanoferrate(III) seems to be less clear; however, tunnelling would most probably occur in that case too. Thus, the point for the reaction in “neat” water fits in the strong linear correlation of ln *A*h/*A*d and ΔΔ*H*^‡^(D,H) that comprise all the systems (see Section 3.5.1.) suggesting the common physical origin of tunnelling in all the cases investigated. Furthermore, the value of ΔΔ*H*^‡^(D,H) that is below the semiclassical limit of 5.1 kJ/mol in that case may be viewed to approach the extensive tunnelling regime. Finally, great negative entropies of activation indicative of tunnelling [81,82], are observed in all the examined reaction systems and the magnitude of the entropy loss observed in the “neat” water reactions suggests a compelling similarity with the values of activation entropy for the great majority of the other systems investigated.

The results of the study of KIE-s and tunnelling in the ascorbate reactions [11,17,18,19,20,21,22,23,72] can contribute to the better understanding of PCET reactions; moreover, many enzymatic reactions involve PCET. The reactions of ascorbate presented are similar to the reactions catalysed by ascorbate peroxidase enzymes [83], and cytochrome b_561_ proteins [84], being thus a good potential reference solution reactions which is of importance for the study of enzyme catalysis.

### 3.1. Reaction of Ascorbate with TEMPO Radical in the Mixed Water-Organic Cosolvent Systems

**Scheme 1:**
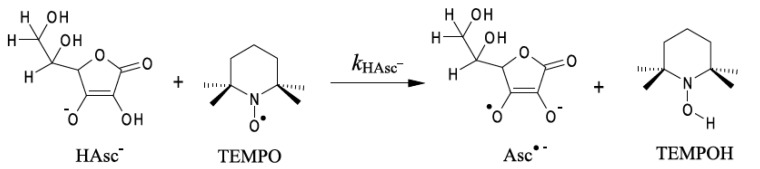
Oxidation of ascorbate monoanion with 2,2,6,6-tetramethylpiperidine-1-oxyl (TEMPO) radical. *k*_HAsc−_ is the second-order rate constant for the rate-controlling proton-coupled electron transfer (PCET) process (first step).

The reaction (Scheme 1) in water as well as in water-organic cosolvent solutions [17,18] involves PCET in the rate-controlling step where hydrogen from 2-OH position of ascorbate is transferred to the TEMPO radical and sizeable isotope effects are observed.

The KIE-s found for various solvent systems are always well beyond the semiclassical limit of 7–10 expected for the hydrogen transfer from an OH moiety, reaching up to *k*h/*k*d of 31 in the water-dioxane solvent system at room temperature (Table 1). The observed isotope effects increase on the decrease of solvent polarity, going from the water reaction solution to the mixed solvents in all the cases examined. This is more obvious in the case of the series of water-dioxane mixtures where the increase of the KIE follows roughly a linear dependence on the molar fraction of dioxane in water [17]. PCET reactions can retain some resemblance with proton transfer reactions and changes of KIE-s (and the tunnelling signatures) on solvent polarity in proton transfer reactions are known for decades [3] but no generally accepted explanation of the issue is available. Theoretical treatments of PCET reactions [13,35,54,73] could explain in part the KIE dependence insofar as solvent polarity may influence DAD or driving force Δ*G*° of the reaction; however, according to the theory, no change of KIE due to variation of the reorganizational energy *λ* will be expected.

The KIE-s well beyond the semiclassical limits observed in the reaction strongly suggest hydrogen tunnelling in the reaction. This is consistent also with the observed isotopic differences in Arrhenius activation parameters *E*_a_(D) − *E*_a_(H) or isotopic differences in activation enthalpy, ΔΔ*H*^‡^(D,H), that are greater than the semiclassically predicted value of 5.1 kJ/mol for the hydrogen transfer from O–H. The finding of this isotopic difference, accompanied by the observed isotopic ratios of Arrhenius prefactors below the semiclassical limit for *A*h/*A*d < 0.5 should be viewed to be indicative of the moderate tunnelling in the reaction (Table 1). However, in the cases of the water-dioxane solution as well as the water solution of tetraethylammonium chloride, the ratios *A*_H_/*A*_D_ could be close to the lower limit of the “extensive” tunnelling where both of the isotopes tunnel or where deuterium begins to tunnel (see however discussion in [17]). In addition, quite significant negative entropies of activation are indicative of attainment of transition configuration well organized for tunnelling [81,82]. Interestingly, the reaction barriers, Δ*G*^‡^, in the reaction performed in quite various solvent systems differ one from another for 1–2 kJ/mol only, while the isotopic ratios of the Arrhenius prefactors suggest significant differences in tunnelling. The isotopic differences in activation enthalpy, ΔΔ*H*^‡^(D,H) greater than 5.1 kJ/mol do not invoke an explanation using the Bell tunnelling correction approach [23,70]. An explanation of the observed tunnelling that will take into account dynamical features of the system following a Marcus-like tunnelling model and the context of ideas on “environmentally assisted tunnelling” [69] has been suggested [18].

### 3.2. Reaction of Ascorbate with Hexacyanoferrate(III) in the Mixed Water-Organic Cosolvent Systems

**Scheme 2:**
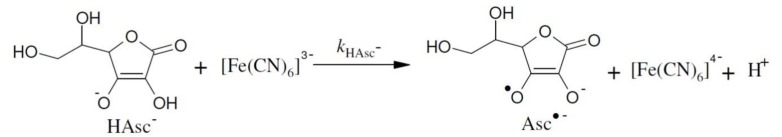
Oxidation of ascorbate monoanion with hexacyanoferrate(III) ion. *k*_HAsc−_ is the second-order rate constant for the rate-controlling PCET process (first step).

In the reaction (Scheme 2) performed in the “neat” water moderate kinetic isotope effect *k*h/*k*d of 4.6 and values *A*h/*A*d of 0.97 and ΔΔ*H*^‡^(D,H) of 3.9 kJ/mol have been observed [19,20].

Surprisingly great changes of the KIE-s and the tunnelling signatures observed have taken place by adding organic cosolvents into water reaction system (Table 2).

Thus, KIE-s in 1:1 water-cosolvent systems are nearly twice the figure in “neat” water system and very pronounced tunnelling signatures, isotopic ratios of the Arrhenius prefactors and the isotopic differences in activation enthalpy are observed. While according to the theory, an increase of KIE-s could be expected on going from the water reaction solution to the mixed solvents employed due to an increase of DAD with the decrease of solvent polarity (when both the reactants are negatively charged, see also above and Section 2.2.), inspection of KIE-s in Table 2 should suggest that other factors also are involved. Namely, the polarity of the water-dioxane and water-ethanol systems surely are lower than that of water-acetonitrile solvent, but the KIE observed in water-acetonitrile is greater than KIE observed in the former two. The theory predicted that KIE-s could increase or decrease owing to a decrease or increase of the reaction driving force Δ*G*°. Therefore, an expectation is, that the addition of the inorganic salts where ion pairs [Fe(CN)_6_]^3−^,K^+^ for example, presumably are formed and driving force should be decreased [23], would lead to an increase of KIE in the reaction. However, the situation is not that simple. In contrast, the addition of 0.1 M KCl lead to the significant decrease of KIE-s in the water-dioxane as well in the water-acetonitrile system (Table 2). More interestingly, the observed tunnelling signatures are essentially of the same magnitude in both the cases. Once again, it seems that explanation of the observed intriguing KIE-s and tunnelling should take into account dynamical features of the system, especially those related also to dynamics of solvation shell; this context could perhaps bear few similarities with the dynamical interactions between the hydration shell and bulk and the protein dynamics [85].

### 3.3. Reaction of Ascorbate with Hexacyanoferrate(III) in More Concentrated Aqueous Solutions of the Salts

Two distinct situations appear with regard to the observations of the KIE-s and hydrogen tunnelling in the reaction in water solutions containing various salts. First, when the solutes added are quaternary ammonium salts, sizeable increases of KIE, even close to the semiclassical limit, and the pronounced hydrogen tunnelling signatures characteristic of moderate tunnelling regime have been observed (Table 3).

Second, with regard to the effects of added organic cosolvents, the effects of the added quaternary ions are very similar in the magnitude and direction of the changes of KIE-s and the tunnelling signatures. However, the conspicuous difference between the two classes of solutes added exists; the observed effects of quaternary ammonium ions appear at concentrations that are one order of magnitude lower than in the case of organic cosolvents. This should probably be a consequence of the formation of (likely solvent separated) ion pairs with the hexacyanoferrate(III) ions; then, organic species could be close to the transition reaction configuration at substantially lower concentrations than in the case of uncharged molecules of the cosolvents. Taking into account the above-described cases of salts added into the mixed solvents (cf. Table 2 in Section 3.2, the decrease of KIE-s due to ions added) however, the open questions about the role of quaternary ions remain, especially because the species are organic having the charge at the same time. Moreover, the case should be questioned also in the light of effects of inorganic ions added into the same reaction water system (see below), including the solvation/desolvation phenomena too.

In the second case, that of inorganic salts added into the water reaction system, quite unexpectedly the decrease of KIE-s and the appearance of hydrogen tunnelling signatures indicative of extensive tunnelling regime have been observed (Table 3). Obviously, only the cations from the inorganic salts added have a role in the observed variation of the KIE-s (and the reaction rates too, see in Table 3); the influence of anions is insignificant. Since these experiments included only the water solutions, the intriguing transition from the moderate hydrogen tunnelling regime to the extensive tunnelling where both the isotopes tunnel could be traced probably in the differences of solvation of both the hexacyanoferrate(III) anion and inorganic cation in water and in the mixed solvents. Indeed, the above-mentioned cases of the decreased KIE-s in the mixed solvents when the cations are present in the reaction solution (see in Table 2), point more to the role of inorganic cations and their solvation/charge density and the differences in ion-ion interactions in the various systems. That is, the interaction with the cations could lead to a more favourable “packaging” of the reactants (due to proximity of cation and the negatively charged transition configuration) thus enabling extensive tunnelling. However, taking into account the tunnelling phenomena observed, there could be one more reason to consider not only bulk solvent properties and ionic effects but dynamical features of the systems as well.

### 3.4. Reaction of Ascorbate with Ferricinium Ions in Aqueous Solutions of Sodium Chloride

The reaction of ascorbate with ferricinium ions [22] involves the interaction of the negatively and the positively charged species. Relative low KIE-s and the ratios of isotopic Arrhenius prefactors signatures consistent with the moderate tunnelling have been observed in the reaction (Table 4). While only few data are available, the fact that observed KIE on addition of the inorganic salt increases and the isotopic differences of activation enthalpies are below the semiclassical limit seemingly appears to be at odds with the results presented above (in Section 3.3.). It would be expected that an interplay of different factors, including these related to the water dynamics, could lead to the peculiarities observed in the case. Relatively small loss of the activation entropy could suggest the existence of tight ion pairs consisted of ascorbate and ferricinium ions in the ground state of reaction. A decrease of the (equilibrium) DAD might be expected due to attractive forces between the oppositely charged reactants. Taken together, the observations would be consistent with well organized transition configuration feasible to hydrogen tunnelling in the process.

### 3.5. Reaction of Ascorbate with Hexacyanoferrate(III) in Fairly Diluted Aqueous Solutions

The study of KIE-s and hydrogen tunnelling phenomena in the above PCET reaction (Scheme 2) performed in fairly diluted water solutions containing low concentrations of various (inert) partially hydrophobic solutes [23] concentrates on the question of the recent proposal of involvement of the collective excitonic vibrational dynamics in aqueous chemistry [24,25,26,27]. The investigation starts from the expectation that these fairly diluted water solutions probably can be considered as a good representation of neat water solutions, since the vibrational excitonic dynamics is critical in neat water [23,25,26]. Furthermore, in the fairly diluted solutions the expectation is that there is no influence of the changes of driving force Δ*G*°, or DAD, the parameters that, according to theory [35,54,73] can influence the magnitude of KIE-s observed in more concentrated water solutions [23]; the only relevant system property change that can be at work in the cases will be related to the changes of water dynamics. These changes occur due to presence of hydrogen-bonded partial hydrophobes added.

**Figure 2:**
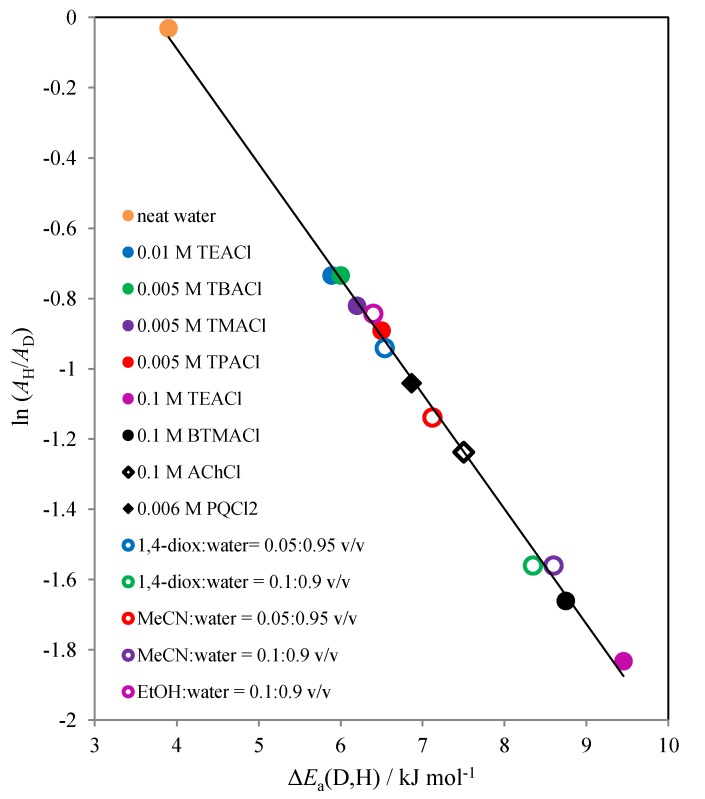
Correlation between Δ*E*_a_(D,H) and ln *A*_H_/*A*_D_. Abbreviations as noted in [23]. Adapted from [23].

The results are surprising; all the KIE-s obtained are increased significantly and the signatures of hydrogen tunnelling indicative of moderate tunnelling are modulated throughout (for more information see Table 1 in [23]). The strongly linear correlation between common tunnelling signatures (Figure 2) points to the common physical origin of the phenomenon in all the cases. In our opinion, the results can be adequately explained by invoking the proposal of the involvement of collective and excitonic vibrational water dynamics in the processes [23].

#### 3.5.1. Reaction of Ascorbate with Hexacyanoferrate(III) in Fairly Diluted Aqueous Solutions: Hydrogen Tunnelling and the Vibrational Water Dynamics Connected?

The explanation [23] of the observed intriguing modulation of the hydrogen tunnelling (and KIE-s) in the reaction in diluted water solutions takes into account the insights obtained from ultrafast 2D IR spectroscopy of water [24,25,26,27,28,29,30,31,32,33]. Here, several points should be noticed. According to the spectroscopy water vibrations are highly coupled and collective in nature. The OH stretch is not only a local vibration but the coherent vibrational motion involving the stretch-bend coupling that can comprise up to six waters. Furthermore, aside from the intermolecular motions mentioned, it was demonstrated [24,26] that the solute and water modes can be correlated when there is H-bonding between the two. Finally, it was proposed that the coherent coupled vibrational motions that involve the solute and water can contribute to activation processes, which should be of profound importance to understanding water chemistry.

**Figure 3:**
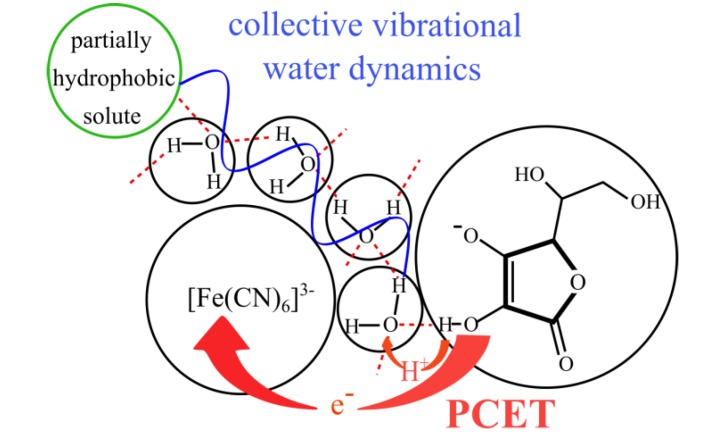
Schematic representation of transition configuration in the PCET ascorbate interaction with hexacyanoferrate(III) ion. Adapted from [23].

The investigated reaction (Scheme 2) may present a good model reaction to test the proposal [24,25,26] as the coherent coupled vibrational motions that involve the solute and water can contribute to the dynamics of transition configuration and the modulation of hydrogen tunnelling in the process. The reaction meets conditions required for the model since there is H-bonding between the transition configuration and water in the concerted reaction. Therefore, since the reaction system involves DAD oscillations, the dynamical feature related to hydrogen tunnelling, a modulation of hydrogen tunnelling should be expected when the partially hydrophobic solute is present in the system leading to changes in the vibrational water dynamics [23]; the experimental findings are in line with the expectation. The transition reaction configuration is presented schematically by Figure 3.

Taken together, the picture is emerging where the water dynamics and tunnelling can be connected. Experimentally, this can be seen through the observed modulation of the hydrogen tunnelling signatures in the reaction, as illustrated by the strongly linear correlation of isotopic values of Arrhenius prefactor ratios ln *A*h/*A*d and isotopic differences in activation enthalpies ΔΔ*H*^‡^(D,H) comprising all the cases of the fairly diluted water systems (Figure 2) [23].

## 4. Conclusions

The most intriguing insights obtained from the studies of KIE-s and the modulation of hydrogen tunnelling phenomena in the PCET reactions of ascorbate should be related to the experiments performed in the diluted water solutions of inert partial hydrophobes; the obtained results should be viewed to confirm the proposal of far-reaching importance [24,26] related to involvement of vibrational water dynamics in activation processes.

The experiments strongly suggest the common physical origin of the phenomenon through the correlation between the obtained hydrogen tunnelling signatures in all the cases.

These insights could perhaps be applied, at least in part and along with the explanations that used the theory of PCET reactions, to the cases of KIE-s and hydrogen tunnelling observed in the reactions in the more concentrated aqueous solutions, as the strong stretch-intermolecular mode coupling, critical for excitonic nature of OH stretch in water, could be seen in a more concentrated salt solution [26].

Not insignificant with regard to the insight in the nature of partial hydrophobes, the investigation of tunnelling in the case described will be of importance for better understanding its role in many situations where hydrophobic species could be relevant to hydrogen transfer/tunnelling process.

## Figures and Tables

**Table 1:** Kinetic isotope effects, activation parameters Δ*H***^‡^** and Δ*S***^‡^**, isotopic differences ΔΔ*H***^‡^**(D,H) and ΔΔ*S***^‡^**(D,H) and ratios *A*h/*A*d in the reaction of ascorbate with TEMPO radical. Adapted from [17,18].

Solvent	KIE	Δ*H*^‡^/kJ mol^−1^	Δ*S*^‡^/JK^−1^ mol^−1^	ΔΔ*H*^‡^/kJ mol^−1^	ΔΔ*S*^‡^/JK^−1^ mol^−1^	*A*h/*A*d
Water	24.2 (0.6)	31.0 (0.4)	−134 (2)	9.0 (0.6)	4 (3)	0.6 (0.2)
Water ^a^	21.9 (0.2)	33.7 (0.4)	−124 (2)	7.0 (0.4)	−2 (2)	1.34 (0.15)
1,4-diox:water ^b^	31.1 (1.1)	23.9 (0.2)	−151 (1)	8.2 (0.4)	−1 (1)	1.2 (0.2)
MeCN:water ^b^	25.4 (0.3)	31.1 (0.1)	−131 (1)	8.4 (0.4)	2 (2)	0.82 (0.1)
MeCN:water ^a,b^	23.3 (0.1)	29.4 (0.3)	−133 (1)	11.6 (0.5)	13 (1)	0.22 (0.03)

^a^ with TEACl 0.5 M; ^b^ organic cosolvent:water = 1:1 *v*/*v*. Abbreviations are as follows: 1,4-dioxane (1,4-diox); acetonitrile (MeCN); tetraethylammonium chloride (TEACl).

**Table 2:** Kinetic isotope effects, activation parameters Δ*H***^‡^** and Δ*S***^‡^**, isotopic differences ΔΔ*H***^‡^**(D,H) and ΔΔ*S***^‡^**(D,H) and ratios *A*h/*A*d in the reaction of ascorbate with hexacyanoferrate(III) Ions in the mixed water-organic cosolvent systems. Adapted from [19,22].

Organic Cosolvent:Water = 1:1 *v*/*v*	KIE	Δ*H*^‡^/kJ mol^−1^	Δ*S*^‡^/JK^−1^ mol^−1^	ΔΔ*H*^‡^/kJ mol^−1^	ΔΔ*S*^‡^/JK^−1^ mol^−1^	*A*h/*A*d
MeCN:water	8.25 (0.09)	24.8 (0.3)	−137.3 (1.0)	6.9 (0.4)	5.9 (1.5)	0.49 (0.09)
MeCN:water ^a^	6.55 (0.05)					
MeCN:water ^b^	6.70 (0.27)					
MeCN:water ^c^	5.86 (0.17)	28.7 (0.2)	−104.0 (0.7)	9.1 (0.3)	16.1 (0.9)	0.14 (0.02)
MeCN:water ^d^	7.81 (0.17)					
1,4-diox:water	7.84 (0.20)	37.6 (0.2)	−99.0 (0.7)	12.7 (0.4)	25.6 (1.4)	0.046 (0.008)
1,4-diox:water ^e^	5.75 (0.14)	35.3 (0.2)	−80.8 (0.8)	10.9 (0.3)	22.1 (1.0)	0.07 (0.01)
1,4-diox:water ^c^	4.39 (0.13)	35.5 (0.3)	−76.5 (1.2)	8.6 (0.6)	16.5 (2.0)	0.14 (0.03)
EtOH:water	7.90 (0.22)	26.4 (0.2)	−136.0 (0.7)	11.9 (0.4)	22.7 (1.2)	0.065 (0.010)
Acetone:water	8.59 (0.13)					

^a^ 0.1 M Na-acetate; ^b^ 0.1 M NaCl; ^c^ 0.1 M KCl; ^d^ MeCN:water = 0.25:0.75 *v*/*v*; ^e^ 0.3 M NaCl. Abbreviations are as follows: 1,4-dioxane (1,4-diox); acetonitrile (MeCN); ethanol (EtOH).

**Table 3:** Kinetic isotope effects, activation parameters Δ*H***^‡^** and Δ*S***^‡^**, isotopic differences ΔΔ*H***^‡^**(D,H) and ΔΔ*S***^‡^**(D,H) and ratios *A*h/*A*d in the reaction of ascorbate with hexacyanoferrate(III) ions in more concentrated aqueous solutions of salts. Adapted from [20,21].

Salt Added	KIE	Δ*H*^‡^/kJ mol^−1^	Δ*S*^‡^/JK^−1^ mol^−1^	ΔΔ*H*^‡^/kJ mol^−1^	ΔΔ*S*^‡^/JK^−1^ mol^−1^	*A*h/*A*d
KCl 0.5 M	3.37 (0.07)	20.3 (0.2)	−121.0 (0.8)	1.8 (0.4)	−4.0 (1.2)	1.61 (0.25)
NaCl 0.5 M	3.78 (0.07)	21.1 (0.4)	−121.2 (1.3)	1.3 (0.6)	−6.9 (2.1)	2.29 (0.60)
NaCl 1.0 M	3.31 (0.07)	21.1 (0.2)	−118.3 (0.6)	1.5 (0.4)	−4.7 (1.3)	1.77 (0.29)
Na-acetate 0.5 M	3.77 (0.12)					
LiCl 0.5 M	3.93 (0.18)					
TEACl 0.5 M	9.49 (0.12)	21.6 (0.3)	−131.0 (1.0)	11.1 (0.5)	19.0 (2.2)	0.10 (0.02)
TEACl 1.0 M	10.08 (0.07)					
TMACl 0.5 M	6.48 (0.08)	15.9 (0.2)	−141.0 (1.0)	6.9 (0.3)	8.0 (1.4)	0.35 (0.06)
TMACl 1.0 M	6.79 (0.13)					
BTMACl 0.5 M	7.53 (0.11)	15.7 (0.2)	−141.8 (0.6)	8.6 (0.2)	12.3 (0.8)	0.23 (0.02)
BTMACl 1.0 M	8.01 (0.19)					

Abbreviations are as follows: tetraethylammonium chloride (TEACl); tetramethylammonium chloride (TMACl); benzyltrimethylammonium chloride (BTMACl).

**Table 4:** Kinetic isotope effects, activation parameters Δ*H***^‡^** and Δ*S***^‡^**, isotopic differences ΔΔ*H***^‡^**(D,H) and ΔΔ*S***^‡^**(D,H) and ratios *A*h/*A*d in the reaction of ascorbate with ferricinium ion. Adapted from [22].

	KIE	Δ*H*^‡^/kJ mol^−1^	Δ*S*^‡^/JK^−1^ mol^−1^	ΔΔ*H*^‡^/kJ mol^−1^	ΔΔ*S*^‡^/JK^−1^ mol^−1^	*A*h/*A*d
Water	1.91 (0.02)	45.0 (0.2)	−26.0 (0.7)	2.1 (0.3)	1.8 (0.9)	0.80 (0.09)
0.5 M NaCl	2.01 (0.03)	45.6 (0.1)	−32.7 (0.4)	3.4 (0.4)	5.5 (1.5)	0.52 (0.10)
MeCN:water ^a^	1.48 (0.03)					

^a^ MeCN:water = 1:1 *v/v*. Abbreviation is as follows: acetonitrile (MeCN).
